# A new stump-toed frog from the transitional forests of NW Madagascar (Anura, Microhylidae, Cophylinae, *Stumpffia*)

**DOI:** 10.3897/zookeys.933.47619

**Published:** 2020-05-18

**Authors:** Angelica Crottini, Gonçalo M. Rosa, Samuel G. Penny, Walter Cocca, Marc W. Holderied, Lovasoa M.S. Rakotozafy, Franco Andreone

**Affiliations:** 1 CIBIO, Research Centre in Biodiversity and Genetic Resources, InBIO Associate Laboratory, Universidade do Porto, Campus Agrário de Vairão, Rua Padre Armando Quintas 7, 4485-661, Vairão, Portugal Universidade do Porto Vairão Portugal; 2 Institute of Zoology, Zoological Society of London, Regent’s Park, NW1 4RY London, UK Institute of Zoology, Zoological Society of London London United Kingdom; 3 Centro de Biologia Ambiental, Faculdade de Ciências da Universidade de Lisboa, Bloco C2, Campo Grande, 1749-016, Lisboa, Portugal Universidade de Lisboa Lisboa Portugal; 4 School of Pharmacy and Biomolecular Sciences, University of Brighton, Brighton BN2 4GJ, UK University of Brighton Brighton United Kingdom; 5 Life Sciences Building, University of Bristol, 24 Tyndall Ave, Bristol BS8 1TQ, UK University of Bristol Bristol United Kingdom; 6 Mention Zoologie et Biodiversité Animale, Faculté des Sciences, Université d’Antananarivo, BP 906, Antananarivo (101), Madagascar Université d’Antananarivo Antananarivo Madagascar; 7 Museo Regionale di Scienze Naturali, Sezione di Zoologia, Via G. Giolitti, 36, I-10123, Torino, Italy Museo Regionale di Scienze Naturali Torino Italy

**Keywords:** Amphibia, Conservation, *Stumpffia froschaueri* sp. nov., UNESCO Sahamalaza – îles Radama Biosphere Reserve, Angorony Forest

## Abstract

A new species of the miniaturised microhylid frog genus *Stumpffia*, from north-western Madagascar, is described. *Stumpffia froschaueri***sp. nov.** differs from all other described *Stumpffia* species in colouration and morphology and is genetically divergent (≥ 7% uncorrected p-distance to all other nominal species of the genus) in a fragment of the mitochondrial 16S rRNA gene and in a segment of the nuclear Rag-1 gene. The new species is reliably known only from a few specimens collected in the Sahamalaza (and surroundings) region. Its known distribution is limited to three forest patches severely threatened by fire, drought and high levels of forest clearance, thus suggesting a classification of “Critically Endangered” according to IUCN Red List criteria.

## Introduction

Madagascar is the fourth largest island and known for its particularly high biodiversity, hosting an exceptional concentration of endemic fauna and flora (de Wit 2003; Wilmé et al. 2006). Large parts of its extant vertebrate fauna result from ancient colonisation events that took place when the island was already separated from all other Gondwanian landmasses (Vences et al. 2003; Crottini et al. 2012).

Although lemurs (currently comprising over 100 species) are Madagascar’s most iconic fauna representatives (Mittermeier et al. 2010), the island is also home to an exceptional diversity of endemic amphibians (Glaw and Vences 2007; Zimkus et al. 2017). Two amphibian species were introduced to Madagascar: the Southeast Asian *Hoplobatrachus tigerinus* (Daudin, 1802) (Kosuch et al. 2001) was introduced historically in north-western Madagascar and has since colonised different habitats and regions (Vences et al. 2003); and the Asian common toad, *Duttaphrynus melanostictus* (Schneider, 1799) introduced in the Toamasina area around 2010 (Andreone et al. 2014a; Crottini et al. 2014) is rapidly expanding its range (Licata et al. 2019).

Most Malagasy amphibian taxa have been described over the course of the last three decades (Glaw and Vences 2007), a period of general increase in the publication of amphibian studies (Vences et al. 2008). Among the native amphibians of Madagascar two groups experienced particularly prolific adaptive radiations: the mantellid frogs (Anura: Mantellidae Laurent, 1946) and the cophyline frogs (Anura: Microhylidae Günther, 1858 (1843): Cophylinae Cope, 1889). The phylogenetic position of the latter is not entirely resolved (e.g., Van der Meijden et al. 2007; De Sá et al. 2012). Cophyline frogs show a high diversity of ecological adaptations (with terrestrial, arboreal, fossorial and rupicolous species; Glaw and Vences 2007) and strong microendemism (Wollenberg et al. 2008; Rakotoarison et al. 2017). They are currently divided into nine genera (Scherz et al. 2016, 2017, 2019). Of these, the genus *Stumpffia* Boettger, 1881 mostly contains small to miniaturised terrestrial species characterised by the absence of maxillary and vomerine teeth (Blommers-Schlösser and Blanc 1991; Köhler et al. 2010), plus a few larger, rock-dwelling species. *Stumpffia* species are generally found in the leaf litter of humid rainforests, although some species occupy the arid north-west (Köhler et al. 2010; Rakotoarison et al. 2017). *Stumpffia* can be nocturnal or diurnal, are generally of cryptic colouration, and wary, ceasing calling when approached (Glaw and Vences 2007). Thus, they are not easy to spot in the field requiring targeted search for their detection. Due to their miniature size and secretive habits, the genus *Stumpffia* was until recently one of the least studied of the Malagasy amphibians, with substantial undescribed diversity (Glaw and Vences 2007; Rakotoasison et al. 2017). A recent revision based on a combination of molecular, bioacoustic, and morphological data, revealed 26 new species (Rakotoarison et al. 2017), now providing the opportunity to further develop the knowledge on this group and other miniaturised frog species of Madagascar (e.g., Scherz et al. 2019).

Four main molecular clades have been identified within the genus *Stumpffia* (Rakotoarison et al. 2017): *i*) Clade A, from northern and north-western Madagascar contains species with limited digital reduction, which has been divided by body size into subclades of large (Subclade A3), small (A1), and miniaturised (A2) species; *ii*) Clade B, from central-east and north-western Madagascar contains some miniaturised species with strong digital reduction and some comparatively large-sized species; *iii*) Clade C, mostly of the eastern rainforest belt of Madagascar (extreme South excluded) contains large as well as small-sized species with moderate digital reduction; and *iv*) Clade D, from north-eastern Madagascar contains two miniaturised species with strong digital reduction.

In this paper we describe a new species of *Stumpffia* belonging to Clade A2 discovered in a limited region of the transitional forest of north-western Madagascar, and provide indications towards establishing its conservation status.

## Materials and methods

### Study site

The Sahamalaza Peninsula is located in the Sofia region, Analalava district, along the north-west coast of Madagascar, more precisely between -14.066S and -14.616S, and 47.866E and 48.066E (Volampeno 2009). In 2007, some portions of the Peninsula were declared the Sahamalaza – Îles Radama National Park, and since 2001 this region is listed within the UNESCO’s network of Biosphere Reserves (Schwitzer et al. 2007). The biosphere reserve contributes to the conservation of three specific habitats: dry semi-deciduous forest, mangrove forest and coral reefs. The forest ecosystem present in this area is one of the remaining patches of dry littoral forest on the west coast; the Bay of Sahamalaza (along a 30 km stretch) delimits a large area of mangrove, and the sea (about 5–10 km west of the Radama Islands) hosts an ancient coral reef. The protected areas comprises approximately 260 km^2^, half of which is marine, protecting the coral reef and the intertidal portion of the mangrove ecosystem, while the other half protects the terrestrial portion of the mangrove ecosystem and the dry semi-deciduous forest fragments (the last one with a total extension of about 75 km^2^). This terrestrial section includes a number of low altitude hillocks with a few seasonal streams on their foothills (Andreone et al. 2001).

These forest patches represent a unique transitional ecosystem with plant species that are typical either for the Sambirano domain or the dry western domain (Ralimanana and Ranaivojaona 1999; Birkenshaw 2004; Schwitzer et al. 2006). Since the Analavory Forest in the north of the peninsula (ca. -14.3833S, 47.9333E) was destroyed by man-made fire in 2004 (Volampeno 2009), two forest blocks remain, which are also increasingly fragmented and suffer strong human pressure: the Anabohazo Forest in the northeeast, which includes the Berara and Anketsakely patches; and the Ankarafa Forest in the west (-14.3833S, 47.7500E) (Schwitzer et al. 2007). These blocks are kept separated by a matrix of savannah and scrubland.

The area has a sub-humid climate with a hotter, wetter season from December to April and a cooler, drier season from May to November (Schwitzer et al. 2007). Monthly mean maximum temperature ranges from 28.5 ± 3.61 °C in July to 39.1 ± 2.11 °C in February; while monthly mean minimum temperature ranges from 13.2 ± 0.81 °C in October to 21.8 ± 0.81 °C in January (Volampeno et al. 2011) and the mean annual precipitation is around 1600 mm (Schwitzer et al. 2007).

Fieldwork took place between January and February 2013 during the hotter and wetter season, when amphibians are expected to be more active (but see also Dubos et al. 2020). Surveys were conducted in Ankarafa Forest (in the west), in Berara and Anketsakely (both included in the Anabohazo Forest in the north-east) and around the village of Betsimipoaka (see fig. 1 in Penny et al. 2017).

**Figure 1.**
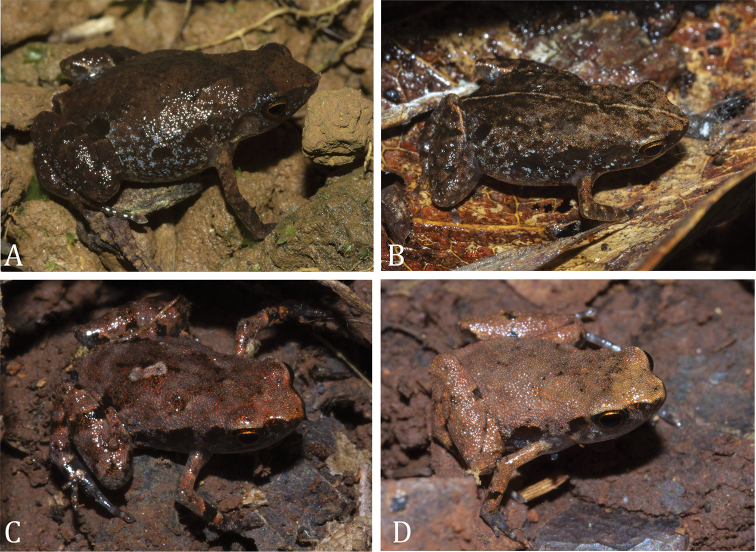
Life colouration of *Stumpffia froschaueri* sp. nov. **A** dorsolateral view of holotype ZSM 169/2019 (ACZCV 0940) from Anketsakely (Anabohazo Forest) **B** dorsolateral view of paratype ZSM 166/2019 (ACZCV 0939) from Ankarafa Forest **C** dorsolateral view of paratype ZSM 168/2019 (ACZCV 0966) from Ankarafa Forest **D** dorsolateral view of paratype ZSM 167/2019 (ACZCV 0968) from Ankarafa Forest. Pictures by Gonçalo M. Rosa

### Voucher specimen collection

Four specimens (Fig. 1) were collected by opportunistic searching during the day and at night (using headlamps and torches at night) and by actively searching in the leaf litter. Voucher specimens were euthanised in MS-222 solution, fixed in 90% ethanol and preserved in 70% ethanol. All individuals were photographed at the time of capture to document life colouration. From each voucher we collected a tissue sample (the fourth toe of the right foot or the whole right foot, depending on specimen size), which was preserved separately in 96% ethanol for genetic analysis. Field numbers ACZCV refer to the collection of A. Crottini.

Locality information were recorded using a GPS receiver (Garmin eTrex Vista HCx; Garmin International Inc., Olathe, United States), datum WGS84. Vouchers were deposited in the Zoologische Staatssammlung München, Munich, Germany (ZSM) (Table 1). Other institutional abbreviations used herein: MRSN, Museo Regionale di Scienze Naturali di Torino, Italy.

### Morphological measurements and description

Morphological measurements (in millimetres) were taken for the four individuals using a dial calliper (Wiha, dialMax) to the nearest 0.1 mm by A.C. (some measurements for the smallest specimen were not taken; Table 1). The following measurements were taken on preserved specimens: SVL, snout-vent length; HW, head width at widest point; HL, head length, measured as the diagonal from the maxillary commissure to the snout tip (Note: this is measured along the jaw, and not parallel to the longitudinal axis of the animal); TD, horizontal tympanum diameter; ED, horizontal eye diameter; END, eye-nostril distance, measured from the anterior corner of eye to the center of the nostril; NSD, nostril-snout tip distance, measured from the centre of the nostril; NND, nostril-nostril distance, measured from the center of the nostrils; FORL, forelimb length, measured from the axilla to the tip of the longest (third) finger with the forelimb extended; HAL, hand length, measured from the base of the hand to the tip of the longest (third) finger; HIL, hindlimb length, measured from the cloaca to the tip of the longest (fourth) toe with the foot extended laterally outward from the body; FOTL, foot and tarsus length, measured from the tibiotarsal articulation to the tip of the longest (fourth) toe; FOL, foot length, measured from the tarsal-metatarsal articulation to the tip of the longest (fourth) toe; TIBL, tibia length, from the tibiotarsal articulation to the knee. Webbing formula follows Blommers-Schlösser (1979).

**Table 1.** Morphometric measurements (in mm) and Institutional Catalogue number of the specimens of *Stumpffia froschaueri* sp. nov. For abbreviations of variables, see methods. Key: F, female; na, not available.

Species	Locality	Catalogue number	Sex	SVL	HW	HL	TD	ED	END	NSD	NND	HAL	FORL	HIL	FOTL	FOL
*Stumpffia froschaueri*	Sahamalaza, Anabohazo Forest, Anketsakely	ZSM 169/2019	F	12.8	4	3.2	1.1	1.9	0.7	1.3	1.5	2.4	5.9	17.6	7.6	4.9
		(ACZCV 0940)														
*Stumpffia froschaueri*	Sahamalaza, Ankarafa Forest	ZSM 167/2019	Undetermined	8.9	3.4	2.6	0.9	1.8	0.7	0.8	1.3	1.6	4.7	12.4	6.1	3.9
		(ACZCV 0968)														
*Stumpffia froschaueri*	Sahamalaza, Ankarafa Forest	ZSM 168/2019	Undetermined	7.6	2.9	2.1	na	1.1	na	na	na	1.3	4.1	9.6	5	3.7
		(ACZCV 0966)														
*Stumpffia froschaueri*	Sahamalaza, Ankarafa Forest	ZSM 166/2019	Undetermined	7.8	2.7	1.8	na	1.4	na	na	na	1.2	4.5	10.9	4.7	2.8
		(ACZCV 0939)														

Terminology follows Vences et al. (2010) and Glaw et al. (2012). Description scheme and definition of body parts used in the description of colour patterns follow Rakotoarison et al. (2017). Description of colour in life is based on the holotype, with some reference to variation as observed in paratypes.

### Molecular analysis

(see Suppl. material 1: Table S1 for GenBank accession numbers and sample information)

Total genomic DNA was extracted using proteinase K digestion (10 mg/ml concentration) followed by a standard salt-extraction protocol (Bruford et al. 1992). We analysed three different markers. A fragment of ca. 550 bp of the 3' terminus of the mitochondrial rrnL (large ribosomal RNA, or 16S rRNA gene; hereafter 3-16S) was already available for the four specimens of this candidate new species (MG189469–MG189472; Penny et al. 2017). These sequences enabled us to assign this mitochondrial lineage to Clade A of the genus *Stumpffia* (sensu Rakotoarison et al. 2017), restricted to the north and north-west of Madagascar. One tissue sample of *S. pygmaea* Vences & Glaw, 1991 (from MRSN A2595) and one of *S. psologlossa* Boettger, 1881 (from MRSN A2594) both from Nosy Be, one tissue samples of *S. huwei* Rakotoarison, Scherz, Glaw, Köhler, Andreone, Franzen, Glos, Hawlitschek, Jono, Mori, Ndriantsoa, Raminosoa, Riemann, Rödel, Rosa, Vieites, Crottini & Vences, 2017 from Montagne d’Ambre and one tissue sample of *S. davidattenboroughi* Rakotoarison, Scherz, Glaw, Köhler, Andreone, Franzen, Glos, Hawlitschek, Jono, Mori, Ndriantsoa, Raminosoa, Riemann, Rödel, Rosa, Vieites, Crottini & Vences, 2017 (from ZSM 204/2016) from Betampona were amplified with primers 16SA-L 5’-CGCCTGTTTATCAAAAACAT-3’ and 16SB-H 5’-CCGGTCTGAACTCAGATCACGT-3’, as described in Crottini et al. (2011). These sequences were generated for comparison and to compile the 3-16S dataset of the *Stumpffia* species that belong to Clade A.

The fragment of ca. 500 bp of the 5’ terminus of the mitochondrial rrnL (large ribosomal RNA, or 16S rRNA gene; 5-16S) has been widely used to assess mitochondrial differentiation in *Stumpffia* (e.g., Köhler et al. 2010; Klages et al. 2013; Rakotoarison et al. 2017). Tissue samples from four specimens of this candidate new species and from one specimen of *S. davidattenboroughi* (from ZSM 204/2016) from Betampona, were amplified using the primers 16SL3 5’-AGCAAAGAHYWWACCTCGTACCTTTTGCAT-3’ and 16SAH 5’-ATGTTTTTGATAAACAGGCG-3’ as described by Vences et al. (2003). A fragment of the nuclear recombination-activating gene 1 (Rag-1) was amplified for the four specimens of this candidate new species with primers Rag1_Coph_F1 5’-CGTGATCGGGTAAAAGGTGT-3’ and Rag1_Coph_R1 5’-TCGATGATCTCTGGAACGTG-3’ as described in (Rakotoarison et al. 2019).

Standard polymerase chain reactions (PCR) were performed in a final volume of 25 μL and using 0.75 μL each of 10 pmol primer, 0.4 μL of total dNTP 10 mM (Promega), 0.1 μL of 5 U/mL GoTaq, 5 μL 5X Green GoTaq Reaction Buffer (Promega) and 4 μl of MgCl_2_ 25mM (Promega). Successfully amplified PCR products were treated to inactivate remaining primers and dNTPs. Purified products were sequenced using dye-labelled dideoxy terminator cycle sequencing on a 3730xl sequencer at Macrogen Inc. Newly generated sequences were checked by eye, edited and aligned in BioEdit (version 7.0.5.3; Hall 1999).

All newly determined sequences were submitted to GenBank (accession numbers 3-16S: MT103416 – MT103419; 5-16S: MT103411 – MT103415; Rag-1: MT090640 – MT090643).

We aligned the 3-16S and 5-16S sequences generated for this study with one sequence for each of the nominal and candidate new species of the *Stumpffia* spp. belonging to Clade A (Rakotoarison et al. 2017). The Rag-1 sequences generated for this study were aligned with all the available Rag-1 sequences of the nominal and candidate new species of the *Stumpffia* spp. of Clade A (Rakotoarison et al. 2017).

Three different datasets were compiled for different purposes: *Dataset 1*, contained all the 3-16S sequences of the *Stumpffia* spp. of Clade A. This alignment contained 25 sequences, excluding only *S. madagascariensis* Mocquard, 1895, *S. sorata* Rakotoarison, Scherz, Glaw, Köhler, Andreone, Franzen, Glos, Hawlitschek, Jono, Mori, Ndriantsoa, Raminosoa, Riemann, Rödel, Rosa, Vieites, Crottini & Vences, 2017 and *S.* sp. Ca30 from Angorony (a forest fragment near Maromandia). It contains and is used to compare the type series (holotype and three paratypes), the nominal, and the candidate species of Clade A. The mean genetic distance matrix (uncorrected p-distance transformed into percent, using the pairwise deletion option) was computed using MEGA, version 7.0.21 (Kumar et al. 2016) (Table 2).

**Figure 2.**
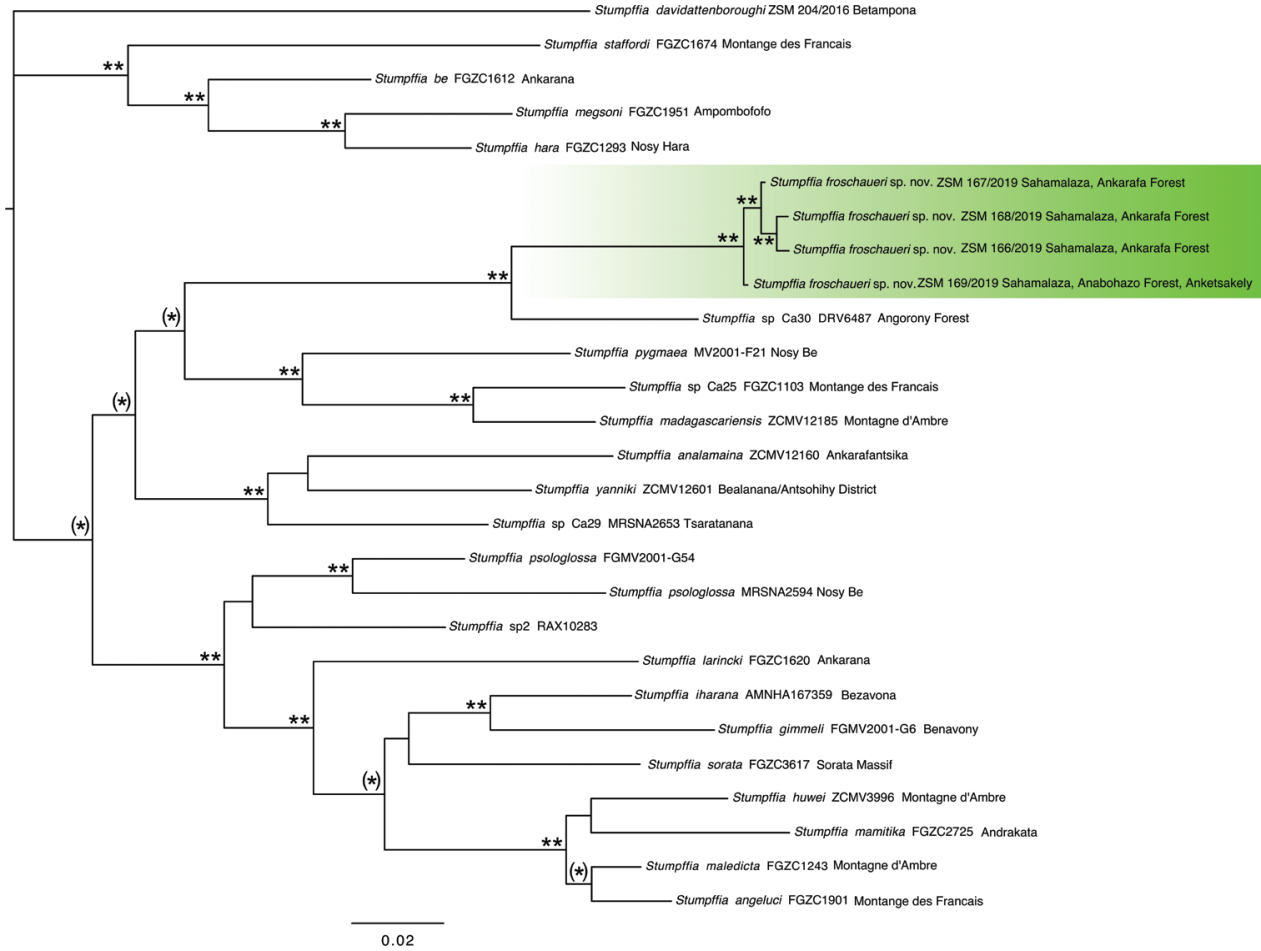
50% majority rule consensus tree; Phylogram from a Bayesian Inference analysis of all the available nominal species and candidate new species of Clade A of the genus *Stumpffia*. Based on 1149 bp of the mitochondrial 3-16S and 5-16S gene fragments. Asterisks mark posterior probabilities: (*) 0.85–0.94, * 0.95–0.98, ** 0.99–1. Scale bar: 0.01 substitutions per site.

*Dataset 2* differs from *Dataset 1* by: *i*) excluding *S. pygmaea*MRSN A2595, *ii*) adding a homologous 3-16S sequence of *S. davidattenboroughi*, and *iii*) including 5-16S sequences for all species except the candidate new species from Andapa - in this study referred to as *S.* sp2 Andapa (voucher: AMNH A181904; present in Peloso et al. 2015). 3-16S and 5-16S sequences of each specimen were concatenated, creating 27 concatenated sequences (length 1149 bp). We used the software Gblocks (Castresana 2000) to exclude highly divergent regions that could not be unambiguously aligned, and used 3-16S and 5-16S homologous sequences of *S. davidattenboroughi* for outgroup rooting (Rakotoarison et al. 2017). We conducted unpartitioned Bayesian inference searches based on the concatenated sequences (Fig. 2). Using the corrected Akaike information criterion (AIC) the best-fitting substitution model was determined in jModelTest2 (Darriba et al. 2012). Unpartitioned Bayesian analyses were conducted in MrBayes 3.2.2 (Ronquist et al. 2012). We performed two runs of 10 million generations (started on random trees) and four incrementally heated Markov chains (using default heating values), sampling the Markov chains at intervals of 1000 generations. Chain mixing, stabilisation and convergence of likelihood values occurred after about 3 million generations. This was inferred examining the standard deviation of split frequencies and by plotting the log likelihoods associated with the posterior distribution of trees in the software TRACER 1.7.1 (Rambaut 2018). The first three million generations were discarded, and seven million trees were retained post burn-in and summed to generate a 50% majority rule consensus tree (Fig. 2).

The purpose of this phylogenetic analysis was: *i*) to show that the two analysed populations of this candidate new species form a monophyletic group; and *ii*) to give a simplified overview of the differentiation of this candidate new species from all other *Stumpffia* species of Clade A, and is not meant to provide an accurate reconstruction of Clade A phylogenetic relationships.

*Dataset 3* contained Rag-1 sequences of the four type specimens and 94 sequences from 18 species of Clade A *Stumpffia*, excluding only *S. megsoni* Köhler, Vences, D’Cruze & Glaw, 2010, *S.* sp2 Andapa and *S. sorata*. We trimmed all sequences to equal length (323 bp) and identified haplotypes using genotype phasing (Stephens et al. 2001) as implemented in the software DnaSP (version 6.12.01; Rozas et al. 2017) with default setting. PHASE algorithm (version 2.1.1) parameters were 1000 iterations, one thinning interval and 100 burn-in iterations and a posterior threshold of 0.9 to determine the most probable inferred haplotypes for each nuclear sequence. Analyses were repeated three times with different seed values. We used the phased sequences to build a ML tree using the Jukes-Cantor substitution model in MEGA7 (Kumar et al. 2016), and entered this tree in Haploviewer (http://www.cibiv.at/~greg/haploviewer) to build a network following the approach of Salzburger et al. (2011) and used this analysis to visualise the occurrence of haplotype sharing in the RAG-1 inferred haplotypes (Fig. 4). This network highlights haplotype similarities between the analysed populations of this candidate new species and differences between the different *Stumpffia* species of Clade A. We interpreted the lack of RAG1 haplotype sharing among individuals of different mitochondrial lineages as an independent indication for their evolutionary independence.

## Results

### Justification for species delimitation

Following Rakotoarison et al. (2017) we used the integration by congruence approach (Padial et al. 2010). This defines species as independent evolutionary lineages if two or more independent lines of evidence support their distinctness.

Firstly, the candidate new species formed a mitochondrial monophyletic group (Fig. 2), with an uncorrected pairwise sequence divergence (p-distance) to other species greater than 3% in the 3-16S gene fragment (Table 2). In amphibians (Fouquet et al. 2007), including Malagasy amphibians (Vieites et al. 2009), this threshold value often corresponds to species-level units.

**Table 2.** Estimates of evolutionary divergence over sequence pairs of Clade A of the genus *Stumpffia* in the analysed 3-16S fragment. The number of base differences per site from averaging over all sequence pairs between groups is shown (p-distance transformed into percent). This analysis involved 24 nucleotide sequences. All ambiguous positions were removed for each sequence pair (pairwise deletion option). There were a total of 528 positions in the final dataset. Pairwise distances calculated for intra- (in bold) and inter-specific genetic divergence. n.c. (not calculated). Highlighted in grey, values above 8%. Analyses were conducted in MEGA 7.0.21 (Kumar et al. 2016).

	*S. froschaueri*	*S. staffordi*	*S. be*	*S. megsoni*	*S. hara*	*S. analamaina*	*S. yanniki*	*S.* sp. Ca29	*S. pygmaea*	*S.* sp. Ca25	*S. psologlossa*	*S. larinki*	*S.* sp2 Andapa	*S. iharana*	*S. gimmeli*	*S. huwei*	*S. mamitika*	*S. maledicta*	*S. angeluci*
*S. froschaueri*	**0.1**%																		
*S. staffordi*	12.3%	**nc**																	
*S. be*	10.8%	7.3%	**nc**																
*S. megsoni*	11.1%	8.5%	3.8%	**nc**															
*S. hara*	11.2%	8.6%	4.9%	3.7%	**nc**														
*S. analamaina*	10.1%	10%	8.3%	8.3%	10%	**nc**													
*S. yanniki*	9.9%	8.1%	7.3%	7.9%	7.8%	5.3%	**nc**												
*S.* sp. Ca29	8.8%	8.4%	7.4%	8.3%	8.4%	5.6%	4.4%	**nc**											
*S. pygmaea*	7.8%	8.5%	7.7%	7.5%	8.7%	7.6%	6.2%	6.6%	**1.1**%										
*S.* sp. Ca25	9.9%	9.8%	8.4%	8.1%	10.3%	7.3%	6.3%	6.8%	5.7%	**nc**									
*S. psologlossa*	11.4%	9.9%	7.9%	9.1%	8.7%	9.5%	7.5%	7.1%	8.6%	9.1%	**4.9**%								
*S. larinki*	10.5%	10.0%	9.1%	9.2%	9.1%	10.0%	8.9%	7.9%	8.3%	10.5%	7.9%	**nc**							
*S.* sp2 Andapa	9.3%	8.4%	7.9%	8.4%	7.9%	8.6%	6.9%	7.2%	7.0%	8.0%	6.0%	8.5%	**nc**						
*S. iharana*	9.1%	9.4%	9.1%	7.9%	9.3%	9.4%	8.4%	8.0%	7.9%	9.4%	7.2%	7.3%	6.9%	**nc**					
*S. gimmeli*	11.2%	10.3%	10.3%	8.5%	9.8%	9.0%	9.0%	9.4%	9.3%	9.0%	8.1%	8.7%	7.4%	4.2%	**nc**				
*S. huwei*	11.1%	10.5%	10.4%	10.4%	11.1%	10.3%	10.0%	8.9%	9.9%	11.0%	8.5%	8.5%	8.5%	5.1%	6.6%	**nc**			
*S. mamitika*	9.6%	9.9%	9.5%	10.5%	9.7%	9.7%	8.7%	8.6%	9.1%	10.6%	7.4%	7.1%	6.9%	6.5%	6.9%	5.5%	**nc**		
*S. maledicta*	8.9%	8.6%	7.7%	8.1%	8.6%	8.3%	7.9%	6.5%	8.2%	9.0%	6.2%	7.5%	6.9%	4.6%	5.3%	2.8%	3.7%	**nc**	
*S. angeluci*	9.3%	9.2%	8.7%	8.6%	9.1%	8.5%	7.7%	7.1%	8.5%	9.9%	6.9%	7.1%	7.1%	4.2%	6.0%	3.5%	5.0%	1.9%	**nc**

Additionally, individuals in this mitochondrial lineage had unique haplotypes of the nuclear Rag-1 gene (Fig. 4), and the concordance between these independent mitochondrial and nuclear markers serves as second line of evidence supporting their distinctness (Avise and Ball 1990).

Finally, like Rakotoarison et al. (2017), we inspected the external morphology of a limited number of traits known to be informative within this genus: (1) body size, (2) externally visible digital reduction, (3) enlarged finger and toe tips, (4) dorsal and ventral colouration, (5) relative length of hands and feet, and (6) the texture of the dorsal skin. Data on advertisement call was not available. The combination of these characters enabled the distinction of the majority of the molecular lineages of *Stumpffia* described in a recent systematic revision of the genus (Rakotoarison et al. 2017). We provide a short diagnosis based on this six-character set only for related species belonging to Clade A1 and A2 (sensu Rakotoarison et al. 2017) and consider the criteria of mitochondrial lineage, unique Rag-1 haplotypes and morphological distinctness to be satisfied when it applies to this set of closely related species.

### Molecular variation and differentiation

The 3-16S fragment analysis (*Dataset 1*; 528 bp) confirms that the four samples of the candidate new species (from two populations) are genetically uniform (intraspecific genetic distance 0.1%; Table 2). The samples of this candidate new species belong to the same mitochondrial lineage (*Dataset 2*; 1149 bp; Fig. 2), which is part of Clade A of the genus *Stumpffia* (Rakotoarison et al. 2017). The genetic distance between this candidate new species and the 18 species and candidate new species of Clade A (for which we had 3-16S data) ranged between 7.8% (with *S. pygmaea*) and 12.3% (with *S. staffordi* Köhler, Vences, D’Cruze & Glaw, 2010) (3-16S data for *S.* sp. Ca30 not available). *S. pygmaea* is currently known only from the island of Nosy Be (ca. 100 km north of Sahamalaza). Smallest pairwise genetic distances were observed between *S. huwei* and *S. maledicta* Rakotoarison, Scherz, Glaw, Köhler, Andreone, Franzen, Glos, Hawlitschek, Jono, Mori, Ndriantsoa, Raminosoa, Riemann, Rödel, Rosa, Vieites, Crottini & Vences, 2017 (2.8%, p-distance) and between *S. maledicta* and *S. angeluci* Rakotoarison, Scherz, Glaw, Köhler, Andreone, Franzen, Glos, Hawlitschek, Jono, Mori, Ndriantsoa, Raminosoa, Riemann, Rödel, Rosa, Vieites, Crottini & Vences, 2017 (1.9%, p-distance). The greatest distance was found between the candidate new species and *S. staffordi* (12.3%). More details on 3-16S genetic distances between species and candidate new species of Clade A of the *Stumpffia* genus are provided in Table 2.

3-16S data for *S.* sp. Ca30 from Angorony Forest (a forest fragment close to Maromandia; -14.22111S, 48.14211E, 115 m a.s.l.) are not available. Therefore, we compared 5-16S data for *S.* sp. Ca30 and the candidate new species here studied. The genetic distance between this candidate new species and *S.* sp. Ca30 (specimen DRV6487; sequence KC351349) at the 5-16S is 8.9%. However, the genetic distance between this candidate new species and specimens DRV6457 and DRV6451 (KC351357 and KC351351, respectively) at the 5-16S is 1.2%, suggesting the latter two specimens belong to the same candidate species here analysed. In view of this finding, we suggest maintaining the use of *S.* sp. Ca30 only for specimen DRV6487, but 3-16S data for all *Stumpffia* specimens collected at Angorony Forest sould be generated to assign the collected specimens to one of these two lineages. Angorony is ca. 30 km north-west from Anketsakely (within Anabohazo Forest) and ca. 50 km from Ankarafa Forest, the two localities where we collected this candidate new species.

In the analysis of *Dataset 2* we included one individual for all nominal species and all published candidate new species of Clade A of the genus *Stumpffia*. The majority rule consensus tree revealed that the four individuals of this candidate new species clustered together, and are the sister group of *S.* sp. Ca30 from Angorony (now restricted to specimen DRV6487). Together, these two mitochondrial lineages have been retrieved (with low support, Posterior probability = 0.91) as the sister clade of *S. pygmaea* + *S. madagascariensis* + *S.* sp. Ca25 (Fig. 2).

The Rag-1 haplotype network (*Dataset 3*; 323 bp; Fig. 4) based on the analyses of 98 specimens from 19 taxa presented a high amount of variation with a total of 88 haplotypes, including a large number of singletons (Fig. 4). Haplotype groups generally coincided with the mitochondrial lineages (compare Figs 2, 4). The inferred Rag-1 haplotypes of the samples from the Sahamalaza Peninsula form one haplogroup (separate by at least three substitutions from all the other nominal species and published candidate species of Clade A). The sample for Angorony Forest for which a Rag-1 sequence was available (DRV 6457; MF768114) is separate by at least seven substitutions from all the other nominal and candidate species of Clade A (Fig. 4). Haplotype sharing among different species was observed only between *S. mamitika* Rakotoarison, Scherz, Glaw, Köhler, Andreone, Franzen, Glos, Hawlitschek, Jono, Mori, Ndriantsoa, Raminosoa, Riemann, Rödel, Rosa, Vieites, Crottini & Vences, 2017 and *S. angeluci*, and between *S. mamitika* and *S. gimmeli* Glaw & Vences, 1992 (Fig. 4).

#### Stumpffia froschaueri sp. nov.

Taxon classificationAnimaliaAnuraMicrohylidae

C5433F0E-4405-59CB-A942-445434192926

http://zoobank.org/97767FBB-9758-441C-895D-FD687C12E111

Figures 1 , 3

##### Notes.

This published work and the nomenclatural acts it contains have been registered in ZooBank, the online registration system for the ICZN. The LSID (Life Science Identifier) for this publication is: urn:lsid:zoobank.org:pub:12D91167-C0F9-4DE2-924A-586A14C62E1D. The electronic edition of this work was published in a journal with an ISSN and has been archived in the following digital repository: https://zookeys.pensoft.net/

##### Remarks.

The species has been previously listed as Stumpffia sp. aff. pygmaea “Sahamalaza” in Penny et al. (2016) and Stumpffia sp. aff. pygmaea Ca “Sahamalaza” (UCS) in Penny et al. (2017), *Stumpffia* sp. 30 in Klages et al. (2013) and *S.* sp. Ca30 in Rakotoarison et al (2017). The latter two names only in the case of specimens DRV6457 and DRV6451, now considered conspecific with *Stumpffia froschaueri* sp. nov. Specimen DRV6487 should continue to be referred as *S.* sp. Ca30.

##### Type-locality.

Anketsakely (Anabohazo Forest, Sahamalaza Peninsula, north-western Madagascar), -14.324712S, 47.910740E; ca 169 m a.s.l., fragment of dry littoral forest included in the buffer zone of the UNESCO Sahamalaza – Îles Radama Biosphere Reserve, G. M. Rosa and L. S. Rakotozafy leg.

##### Material examined.

**Figure 3.**
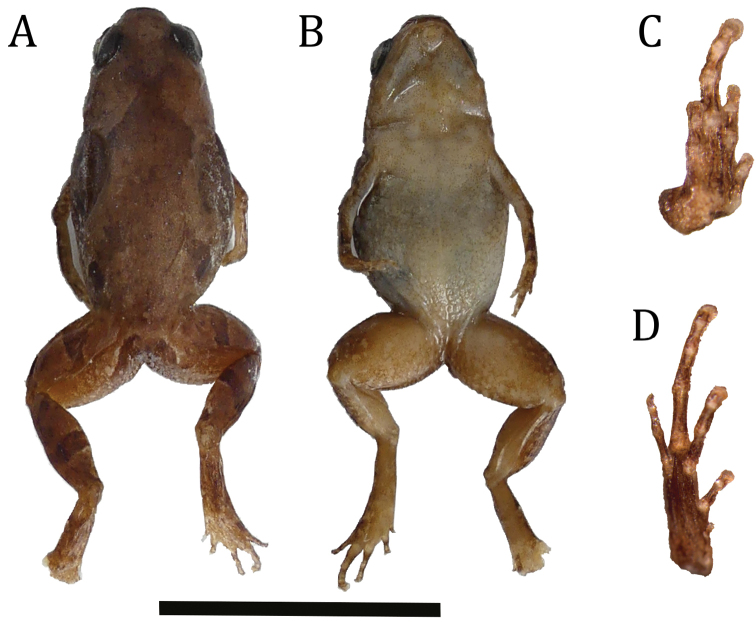
Voucher colouration of *Stumpffia froschaueri* sp. nov. **A** dorsal and **B** ventral views of the preserved holotype of *Stumpffia froschaueri* (ZSM 169/2019; ACZCV 0940), and ventral surfaces of **C** hand and **D** foot. Scale bars: 10 mm; hand and foot not to scale.

***Holotype*.**ZSM 169/2019 (ACZCV 0940) (Fig. 1A and Fig. 3), adult female collected on 30 January 2013 at Anketsakely (Anabohazo Forest, Sahamalaza Peninsula, north-western Madagascar), -14.324712S, 47.910740E; ca 169 m a.s.l., fragment of dry littoral forest included in the buffer zone of the UNESCO Sahamalaza – Îles Radama Biosphere Reserve, G. M. Rosa and L. S. Rakotozafy leg.

***Paratypes*.**ZSM 167/2019 (ACZCV 0968) (Fig. 1D), subadult undetermined collected on 23 January 2013 at Ankarafa Forest (Sahamalaza Peninsula, north-western Madagascar), -14.376367S, 47.761817E; ca 191 m a.s.l.; transitional forest, by G. M. Rosa and L. S. Rakotozafy; ZSM 168/2019 (ACZCV 0966) (Fig. 1C), juvenile undetermined collected on 23 January 2013 at Ankarafa Forest (Sahamalaza Peninsula, north-western Madagascar), -14.376441S, 47.761838E; ca 193 m a.s.l.; transitional forest, by G. M. Rosa and L. S. Rakotozafy; ZSM 166/2019 (ACZCV 0939) (Fig. 1B), juvenile undetermined, collected on 24 January 2013 at Ankarafa Forest (Sahamalaza Peninsula, north-western Madagascar), -14.376241S, 47.761224E; ca 211 m a.s.l.; transitional forest, by G. M. Rosa and L. S. Rakotozafy.

##### Diagnosis.

A species assigned to the small-sized/miniaturised species of Clade A (Clade A1 + A2) of the genus *Stumpffia* based on the small size, absence of digital reduction, absence of enlarged discs on fingers and toes, occurrence in the north-west of Madagascar. The species is placed in Clade A2, which contains four nominal species (*S. madagascariensis*, *S. pygmaea*, *S. yanniki* Rakotoarison, Scherz, Glaw, Köhler, Andreone, Franzen, Glos, Hawlitschek, Jono, Mori, Ndriantsoa, Raminosoa, Riemann, Rödel, Rosa, Vieites, Crottini & Vences, 2017, *S. analamaina* Klages, Glaw, Köhler, Müller, Hipsley & Vences, 2013), most similar to *S. pygmaea* and *S. analamaina* but strongly divergent in mitochondrial and nuclear DNA from these species (and see differential diagnosis below). Although we lack bioacoustic data for this taxon, we here suggest its status as new species due to the high genetic differentiation from all other species in Clade A (pairwise 16S distance ranging from 7.8% to 12.3%), a lack of haplotype sharing in the Rag-1 analysed fragment, and a combination of morphological characters: (1) miniature to small-sized species (SVL 8.9–12.8 mm); (2) manus with four fingers (not obviously reduced in length) and pes with five toes (first toe slightly reduced in length); (3) terminal phalanges of fingers and toes without enlarged discs; (4) relative hand and foot length, HAL/SVL 0.18–0.19, FOTL/SVL 0.59–0.69; (5) dorsum smooth or very slightly tubercular; (6) brownish colouration with indistinct pattern and without contrasted ventral colouration, red colour elements on ventral side, or sharp colour border between dorsum and flanks, presence of darker blotches in the lateral portion.

Distinguished from *S. be* Köhler, Vences, D’Cruze & Glaw, 2010, *S. hara* Köhler, Vences, D’Cruze & Glaw, 2010, *S. megsoni*, *S. staffordi*, *S. meikeae* Rakotoarison, Scherz, Glaw, Köhler, Andreone, Franzen, Glos, Hawlitschek, Jono, Mori, Ndriantsoa, Raminosoa, Riemann, Rödel, Rosa, Vieites, Crottini & Vences, 2017, *S. roseifemoralis* Guibé, 1974, *S. nigrorubra* Rakotoarison, Scherz, Glaw, Köhler, Andreone, Franzen, Glos, Hawlitschek, Jono, Mori, Ndriantsoa, Raminosoa, Riemann, Rödel, Rosa, Vieites, Crottini & Vences, 2017, *S. achillei* Rakotoarison, Scherz, Glaw, Köhler, Andreone, Franzen, Glos, Hawlitschek, Jono, Mori, Ndriantsoa, Raminosoa, Riemann, Rödel, Rosa, Vieites, Crottini & Vences, 2017, *S. diutissima* Rakotoarison, Scherz, Glaw, Köhler, Andreone, Franzen, Glos, Hawlitschek, Jono, Mori, Ndriantsoa, Raminosoa, Riemann, Rödel, Rosa, Vieites, Crottini & Vences, 2017, *S. pardus* Rakotoarison, Scherz, Glaw, Köhler, Andreone, Franzen, Glos, Hawlitschek, Jono, Mori, Ndriantsoa, Raminosoa, Riemann, Rödel, Rosa, Vieites, Crottini & Vences, 2017, *S. edmondsi* Rakotoarison, Scherz, Glaw, Köhler, Andreone, Franzen, Glos, Hawlitschek, Jono, Mori, Ndriantsoa, Raminosoa, Riemann, Rödel, Rosa, Vieites, Crottini & Vences, 2017, *S. fusca* Rakotoarison, Scherz, Glaw, Köhler, Andreone, Franzen, Glos, Hawlitschek, Jono, Mori, Ndriantsoa, Raminosoa, Riemann, Rödel, Rosa, Vieites, Crottini & Vences, 2017, *S. jeannoeli* Rakotoarison, Scherz, Glaw, Köhler, Andreone, Franzen, Glos, Hawlitschek, Jono, Mori, Ndriantsoa, Raminosoa, Riemann, Rödel, Rosa, Vieites, Crottini & Vences, 2017, *S. analanjirofo* Rakotoarison, Scherz, Glaw, Köhler, Andreone, Franzen, Glos, Hawlitschek, Jono, Mori, Ndriantsoa, Raminosoa, Riemann, Rödel, Rosa, Vieites, Crottini & Vences, 2017, *S. grandis* Guibé, 1974 and *S. kibomena* Glaw, Vallan, Andreone, Edmonds, Dolch & Vences, 2015 by smaller body size (8.9–12.8 mm vs. 14.4–27.9 mm); from *S. miery* Ndriantsoa, Riemann, Vences, Klages, Raminosoa, Rödel & Glos, 2013, *S. davidattenboroughi*, *S. tridactyla* Guibé, 1975, *S. contumelia* Rakotoarison, Scherz, Glaw, Köhler, Andreone, Franzen, Glos, Hawlitschek, Jono, Mori, Ndriantsoa, Raminosoa, Riemann, Rödel, Rosa, Vieites, Crottini & Vences, 2017, *S. tetradactyla* Vences & Glaw, 1991, *S. makira* Rakotoarison, Scherz, Glaw, Köhler, Andreone, Franzen, Glos, Hawlitschek, Jono, Mori, Ndriantsoa, Raminosoa, Riemann, Rödel, Rosa, Vieites, Crottini & Vences, 2017, *S. obscoena* Rakotoarison, Scherz, Glaw, Köhler, Andreone, Franzen, Glos, Hawlitschek, Jono, Mori, Ndriantsoa, Raminosoa, Riemann, Rödel, Rosa, Vieites, Crottini & Vences, 2017, *S. betampona* Rakotoarison, Scherz, Glaw, Köhler, Andreone, Franzen, Glos, Hawlitschek, Jono, Mori, Ndriantsoa, Raminosoa, Riemann, Rödel, Rosa, Vieites, Crottini & Vences, 2017, *S. dolchi* Rakotoarison, Scherz, Glaw, Köhler, Andreone, Franzen, Glos, Hawlitschek, Jono, Mori, Ndriantsoa, Raminosoa, Riemann, Rödel, Rosa, Vieites, Crottini & Vences, 2017, *S. miovaova* Rakotoarison, Scherz, Glaw, Köhler, Andreone, Franzen, Glos, Hawlitschek, Jono, Mori, Ndriantsoa, Raminosoa, Riemann, Rödel, Rosa, Vieites, Crottini & Vences, 2017, *S. spandei* Rakotoarison, Scherz, Glaw, Köhler, Andreone, Franzen, Glos, Hawlitschek, Jono, Mori, Ndriantsoa, Raminosoa, Riemann, Rödel, Rosa, Vieites, Crottini & Vences, 2017 and *S. garaffoi* Rakotoarison, Scherz, Glaw, Köhler, Andreone, Franzen, Glos, Hawlitschek, Jono, Mori, Ndriantsoa, Raminosoa, Riemann, Rödel, Rosa, Vieites, Crottini & Vences, 2017 by a lower degree of digital reduction. Differs from most species in Clade A1 (*S. angeluci*, *S. gimmeli*, *S. huwei*, *S. iharana* Rakotoarison, Scherz, Glaw, Köhler, Andreone, Franzen, Glos, Hawlitschek, Jono, Mori, Ndriantsoa, Raminosoa, Riemann, Rödel, Rosa, Vieites, Crottini & Vences, 2017, *S. larinki* Rakotoarison, Scherz, Glaw, Köhler, Andreone, Franzen, Glos, Hawlitschek, Jono, Mori, Ndriantsoa, Raminosoa, Riemann, Rödel, Rosa, Vieites, Crottini & Vences, 2017, *S. mamitika*, *S. maledicta* and *S. sorata*) by slightly smaller body size (8.9–12.8 mm vs. 11–18.1 mm).

Distinguished from *S. psologlossa* (the type species of the genus *Stumpffia*) by manus with first finger not reduced in length (vs. slightly reduced), dorsum smooth (vs. tubercular), different colour pattern (absence of distinct dark brown patches on the back; absence of the brown bands along the flanks). Different from *S. analamaina* by manus with first finger not reduced in length (vs. slightly reduced) and smaller relative hand length (HAL/SVL 0.18–0.19 vs. HAL/SVL 0.20–0.24). Distinguished from *S. gimmeli* by smaller size (SVL 8.9–12.8 mm vs. adult male SVL 14.5 mm), manus with first finger not reduced in length (vs. slightly reduced), pes with first toe slightly reduced in length (vs. first toe almost not reduced in length), terminal phalanges of fingers and toes without enlarged discs, smaller relative hand length (HAL/SVL 0.18–0.19 vs. HAL/SVL 0.19–0.23), dorsum smooth (vs. tubercular), colour pattern (absence of yellow colour on the abdomen vs. presence). Differs from *S. madagascariensis* by manus with first finger not reduced in length (vs. slightly reduced), pes with first toe slightly reduced in length (vs. first toe strongly reduced in length), not enlarged terminal phalanges of toes (vs. slightly enlarged), larger relative hand length (HAL/SVL 0.18–0.19 vs. HAL/SVL 0.15–0.18), dorsum smooth (vs. tubercular), a different colour pattern (absence of sharp colour border between lighter dorsum and darker flanks vs. presence). Distinguished from *S. pygmaea* by the first finger not reduced in length (vs. slightly reduced) and a different colour pattern (presence of indistinct dorsal patter vs. absence; presence of darker blotches in the lateral portion vs. absence). Different from *S. angeluci* by smaller size (SVL 8.9–12.8 mm vs. SVL 13.7–16.1 mm), terminal phalanges of toes without enlarged discs (vs. slightly enlarged discs), smaller relative hand length (HAL/SVL 0.18–0.19 vs. HAL/SVL 0.20–0.25), dorsum smooth (vs. slightly tubercular), colour (dorsal brownish vs. apricot; ventrally absence of yellow colour on the abdomen vs. presence). Distinguished from *S. huwei* by smaller size (SVL 8.9–12.8 mm vs. SVL 12.5–14.8), terminal phalanges of toes without enlarged discs (vs. slightly enlarged discs), colour (dorsally brownish vs. greyish to reddish brown; ventrally cream vs. yellowish) and colour pattern (presence of darker blotches in the lateral portion vs. absence). Differs from *S. iharana* by smaller size (SVL 8.9–12.8 mm vs. SVL 14.0–15.5 mm), terminal phalanges of toes without enlarged discs (vs. slightly to moderately enlarged discs), dorsum smooth (vs. smooth with few scattered tubercles), colour (ventrally cream vs. yellowish) and colour pattern (presence of darker blotches in the lateral portion vs. absence). Distinguished from *S. larinki* by terminal phalanges of fingers and toes without enlarged discs (vs. slightly to moderately enlarged discs), smaller relative hand length (HAL/SVL 0.18–0.19 vs. HAL/SVL 0.22–0.24), colour (brownish vs. iridescent copper) and colour pattern (presence of darker blotches in the lateral portion vs. absence; ventrally uniform cream vs. presence of yellow blotches). Differs from *S. maledicta* by smaller size (SVL 8.9–12.8 mm vs. SVL up to 16.8 mm), manus with first finger not reduced in length (vs. weakly reduced), pes with first toe slightly reduced in length (vs. distinctly reduced), terminal phalanges of toes without enlarged discs (vs. slightly enlarged discs), dorsum smooth (dorsum slightly to moderately tubercular), colour (ventrally cream vs. translucent lemon yellow) and colour pattern (indistinct pattern vs. uniform colour; presence of darker blotches in the lateral portion vs. absence). Distinguished from *S. mamitika* by smaller size (SVL 8.9–12.8 mm vs. male SVL 12.7–15.0 mm), manus with first finger not reduced in length (vs. slightly reduced), terminal phalanges of toes without enlarged discs (vs. slightly enlarged discs), dorsum smooth (vs. smooth with few scattered tubercles or slightly tubercular), colour (dorsally brownish vs. russet). Distinguished from *S. sorata* by smaller size (SVL 8.9–12.8 mm vs. SVL 15.6–16 mm), manus with first finger not reduced in length (vs. slightly reduced), terminal phalanges of toes without enlarged discs (vs. slightly enlarged discs), dorsum smooth (vs. slightly to moderately granular), colour (dorsally brownish vs. taupe) and colour pattern (ventrally uniform cream vs. presence of yellow blotches). Different from *S. yanniki* by manus with first finger not reduced in length (vs. moderately reduced), pes with first toe slightly reduced in length (vs. distinctly reduced); colour pattern (indistinct pattern vs. well-contrasted central dark teddy bear-shaped middorsal marking; presence of darker blotches in the lateral portion vs. absence).

##### Description of the holotype

ZSM 169/2019, female (Figs 1A, 3). Specimen in good state of preservation, third, fourth and fifth toes of the left foot removed as a tissue sample for DNA extraction. Body roundish; head wider than long, narrower than body; snout rounded in dorsal view, slightly pointed in lateral view; nostrils directed laterally, not protuberant, nearer to tip of snout than to eye; canthus rostralis straight; loreal region straight and slightly oblique; tympanum distinct, about 58% of eye diameter; supratympanic fold slightly visible; tongue broadening posteriorly, ending slightly pointy, attached anteriorly, not notched; maxillary teeth and vomerine teeth absent; choanae round. Forelimbs slender; subarticular tubercles single, distinct; outer metacarpal tubercle distinct, single, oval; palmar tubercle distinct, single, oval, smaller in size to outer metacarpal tubercle; inner metacarpal tubercle, slightly smaller than the other carpal tubercles; fingers without webbing; no fingers reduced; relative length of fingers 1 < 4 < 2 < 3; finger tips not expanded into discs. Hind limbs slightly slender; tibio-tarsal articulation reach tympanum when adpressed forward along the body, TIBL 38% of SVL; lateral metatarsalia strongly connected; inner metatarsal tubercle distinct, small, and oval; outer metatarsal tubercle absent; no webbing between toes; toes not reduced; relative length of toes1 < 2 < 5 < 3 < 4; fifth toe distinctly shorter than third. Skin on dorsum smooth, without distinct dorsolateral folds; ventral skin smooth.

##### Colouration of the holotype

ZSM 169/2019, female (Fig. 3). After six years in 70% ethanol red-brownish colouration with indistinct darker markings. Two darker dots are visible over the anterior of the scapular region (above eye), forming the anterior ends of a faint X-like marking above the scapulae. Flanks with the same colour of the dorsum but with several small cream flecks. Four dark blotches are present in the lateral portion: first blotch on tympanum, two irregular blotches between arm and legs insertion, and a large, roundish blotch on the inguinal region. A darker blotch is present also in cloaca region. Nostril indistinctly surrounded by brown; lateral head same colour as dorsum. Abdomen and pectoral region cream, flecked with brownish spots, which become more abundant on chin and ventral surface of thigh; ventral shank uniform brown; sole of foot brown, lighter brown in correspondence to the subarticular tubercles; dorsal thigh brown as dorsum, with a defined darker brown crossband; dorsal shank brown, with a defined perpendicular darker brown crossband midway along its length, and in lateral view with several small cream flecks; posterodorsal surface of shank brown; dorsal foot brown with two slightly defined perpendicular darker brown crossbands dividing the foot in three segments of equal size; toes mottled brown. Arms dorsally light brown with darker (brownish) irregular flecks that become more abundant in the lower arm; hands speckled.

##### Colour in life

of the holotype ZSM 169/2019, female (Fig. 1). Dorsum burnt umber with undefined dark brown markings (Fig. 1A). Slightly defined interocular bar, markings in suprascapular region forming a X-shaped marking, a weak anterior chevron from the inguinal region to the mid-back (Fig. 1A). Flank with multiple cream flecks that become increasingly cream ventrally. Cream flecks present also in lateral head. Four large ebony patches: one less distinct patch runs from the posterior margin of the eye, curving toward the anterior insertion of the arm over the tympanum, two posteriorly to the arm insertion (Fig. 1A) and one in the inguinal region (Fig. 1). One ebony spot in cloacal region (Fig. 1A). Dorsal forelimbs dark orange, with irregular brown markings, forearm brown with two brown crossbands (Fig. 1A). The fingers are mottled brown and cream (Fig. 1A). The dorsal legs are as the back, with one crossband at the mid-thigh and one on the mid-shank (Fig. 1A). Lower dorsal shank with several small cream flecks. The toes are mottled ebony and brown (Fig. 1A). Ventral skin colouration in life unknown. The iris of the holotype is copper reticulated with black, becoming metallic red close to the anterior and posterior corner of the pupil.

##### Variation.

Dorsum can be light brown (Fig. 1C, D) and have a dark cream vertebral line (Fig. 1B). Dorsal markings can be more irregular (Fig. 1B, D). Lateral cream flecks can be present also on tympanum (Fig. 1B). The lateral ebony spots can be less defined and be partially fused (cf. Fig. 1B–D). Toes are mottled ebony and brown and the fourth toe can have a white annulus before the terminal phalange (Fig. 1B).

For variation in measurements among specimens, see Table 1. ZSM 168/2019 (ACZCV 0966) and ZSM 166/2019 (ACZCV 0939), too small to be measured for all measurements. All examined specimens agree strongly with the holotype (although they are distinctly smaller in size) in hand and feet morphology, in having a smooth or very slightly tubercular dorsum and on the presence of an ebony spot over the tympanum, in the inguinal region and in cloacal region. Colour and colour pattern is variable. The degree of visibility of hindlimb crossbands varies strongly, but they are present to some degree in all specimens.

ZSM 167/2019 (ACZCV 0968) (Fig. 1D) is grey on dorsum with a few dark brown markings, absence of the X-marking on suprascapular region, the two blotches between the arm and leg insertions are fused, colourations on arm, hand, legs and feet less dark and markings less distinct (crossbands only slightly distinct), tympanum distinct, ca. 50% of eye diameter; ZSM 168/2019 (ACZCV 0966) (Fig. 1C) dorsally dark brownish, the two blotches between the arm and leg insertions are fused, abdomen, pectoral region and chin darker (with more brown flecks), tympanum distinct, approximately the same size of the eye; ZSM 166/2019 (ACZCV 0939) (Fig. 1B) dorsally brown-greyish, with a greyish vertebral line, the two blotches between the arm and leg insertions are fused, abdomen, pectoral region and chin darker (with more brown flecks).

##### Etymology.

The species name is a patronym in the genitive case, honouring Christoph Froschauer (ca. 1490 – April 1564). His family name means “the man from the floodplain full of frogs”. Froschauer was the first, and European wide renowned, printer in Zürich and he used to sign his books with a woodcut showing frogs under a tree in a landscape. He was notably known for printing Conrad Gessner’s encyclopaedic “*Historia animalium*” and the “Zürich Bible”, a complete translation into German of the Bible several years before Luther’s Bible appeared. Froschauer published works by Zwingli, Bullinger, Gessner, Erasmus von Rotterdam and Luther during his lifetime. His activity represents the nucleus of the Orell Füssli publishing house, which celebrated its 500^th^ birthday on 9^th^ November 2019, which is the date he was given citizenship in Zürich as a gift for his art.

##### Distribution, conservation and proposed IUCN Red List status.

This species is known only from north-western Madagascar and apparently restricted to three forest blocks embedded in a matrix of highly degraded habitat: 1) Anketsakely (within Anabohazo Forest block), 2) Ankarafa Forest, and 3) Angorony Forest. The latter locality is assigned to this species based on the DNA sequences deposited in GenBank (accession numbers KC351357 and KC351351) that correspond to specimens DRV6457 and DRV6451 (not examined by us). This forest fragment lays in close proximity to Sahamalaza Peninsula and it is ca. 30 km away from Anketsakely. The range encompasses elevations from 100–340 m above sea level. The suggested conservation status was assessed using the guidelines of the IUCN Red List (IUCN Standards and Petitions Subcommittee 2019). If suitable habitat is considered to be all areas of Ankarafa Forest, Anabohazo Forest (where Anketsakely lies; likely an over-estimate) and Angorony Forest, then the EOO (extent of occurrence) totals 246 km^2^. If plots with a scale of 2 km^2^ are used to estimate AOO (area of occupancy), then this species occurs within 6 km^2^ of habitat. Similar to the recently described *Boophis ankarafensis* (Penny et al. 2014) and the other microendemic species of the Sahamalaza peninsula (e.g., *Cophyla berara*), this species is likely to be restricted to the typical transitional forest present in this area. Although two of these forest patches are now part of a UNESCO Biosphere Reserve (the Sahamalaza-Îles Radama Biosphere Reserve), forest border patrolling is lacking and forest is still under strong pressure from slash-and-burn activities and timber harvesting (Penny et al. 2016). Furthermore, the species’ apparent preference for intact forest is likely to limit gene flow between the three known populations. Thus, it is important to establish whether the distribution of this species occurs outside these areas. Habitat loss and fragmentation of these forest fragments is likely the greatest threat to this species’ survival, as indicated by the destruction of the nearby Analavory Forest in 2004 after a man-made fire. Given this on-going destruction of suitable habitat, population declines can be expected to continue unless some remedial action is taken. Thus the species should qualify as Critically Endangered under criterion B (B2ab (i, ii, iii, iv, v) of the IUCN Red List (IUCN Standards and Petitions Subcommittee 2019).

##### Natural history.

In Anketsakely and Ankarafa this species has been found only in areas with relatively undisturbed forest. Active individuals were found during the day within the leaf-litter on the forest floor, where discreet calling males were also detected.

##### Call.

Unavailable for analysis. The call of this species is quite inconspicuous and very difficult to locate (G.M. Rosa pers. obs.).

**Figure 4.**
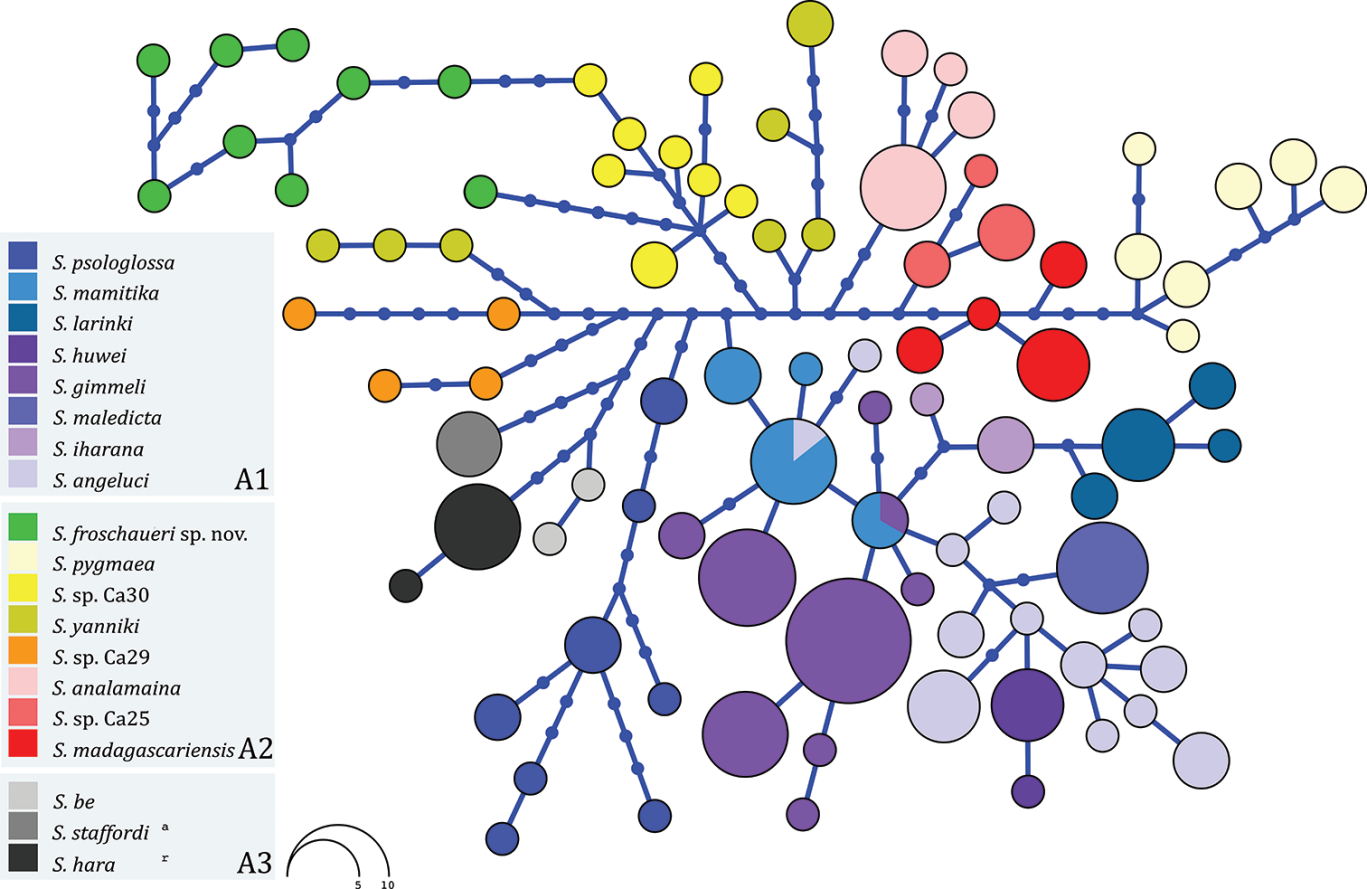
Haplotype network reconstruction (based on 323 bp, haplotypes inferred using the Phase algorithm); all available Rag-1 sequences for the nominal and candidate new species of Clade A of the genus *Stumpffia* (*sensu*Rakotoarison et al. 2017) were used. Small dots represent unsampled or extinct haplotypes, whereas bars represent mutational steps. Overlapping Rag-1 fragment of *Stumpffia megsoni*, *S. sorata* and *S.* sp. Ca07 were not available.

## Discussion

Traditionally the individuals belonging to the terrestrial genus *Stumpffia* were all attributed to a few species described in historical times using morphological patterns. These studies were limited by the frogs’ small size, shy habits and overall morphological similarities limiting further research (Glaw and Vences 2007). However, a systematic revision of the genus in 2017 identified a set of diagnostic morphological characters, and through the use of an integrative taxonomic approach (that combined morphology, genetics and bioacoustics) expanded the number of described species within the genus *Stumpffia* from 15 to 41 (Rakotoarison et al. 2017). The description of *Stumpffia froschaueri* adds a further new species to this genus. This description confirms, once more, that the list of the endemic herpetofauna of Madagascar, although object of intensive and ongoing research activity in the last decades, is still far from being completed, and suggests that field research is still playing a key role in new species discoveries. In fact, *S. froschaueri* was first identified only in the survey that took place in 2013 and went unnoticed in all previous herpetological surveys that took place in the area (Andreone et al. 2001; Raselimanana 2008; Penny et al. 2017).

The residual and threatened forests of Sahamalaza and surrounding areas lie off the touristic routes of Madagascar, and although a few herpetological inventories have been conducted in the area, a full understanding of its biodiversity composition is yet to be achieved. Several candidate species from the area require assessment and, if needed, description (e.g., *Blommersia* sp. Ca05, *Platypelis* sp., *Rhombophryne* sp. among amphibians; and Geckolepis sp. aff. maculata and Phelsuma sp. aff. quadriocellata among the squamata). Previous field surveys contributed to considerable advances in the herpetological knowledge of an otherwise quite unknown area, which is mostly known for the presence of microendemic and highly threatened lemur fauna. These surveys already resulted in the description of one skink (*Pseudoacontias menamainty*), two treefrogs (*Boophis tsilomaro* and *B. ankarafensis*) and a cophyline frog (*Cophyla berara*). All of which are currently known only from this small area of transitional forest and therefore, immediately proposed to be assessed in the higher threatened categories of the IUCN red listing framework (IUCN 2019). *Stumpffia froschaueri* is yet another new species thus far known only from this limited area of north-western Madagascar characterised by the presence of a dry semi-deciduous forest.

In Madagascar, more than 45% of currently described amphibian species (365 taxa) are considered under severe threat of extinction (IUCN 2019). Considering also that the list of candidate new species is estimated to contain ca. 200 more taxa (Vieites et al. 2009; Perl et al. 2014) it is evident that the discovery of a small and inconspicuous frog species with a limited distribution like the currently described *Stumpffia* deserves important attention. We here reiterate the need to continue with field survey activities, giving particular attention to small and marginal areas, where several microendemic candidate species are likely waiting to be discovered. This description confirms the Sahamalaza Peninsula as an important hotspot of amphibian diversity, with several threatened species relying almost entirely on the persistence of these residual forest fragments.

## Supplementary Material

XML Treatment for Stumpffia froschaueri

## Supplementary materials

Supplementary material 1 Table S1 Data type Microsoft Excel Worksheet (.xlsx) Explanation note GenBank accession numbers and sample information. https://binary.pensoft.net/file/411444This dataset is made available under the Open Database License (http://opendatacommons.org/licenses/odbl/1.0/). The Open Database License (ODbL) is a license agreement intended to allow users to freely share, modify, and use this Dataset while maintaining this same freedom for others, provided that the original source and author(s) are credited.Angelica Crottini, Gonçalo M. Rosa, Samuel G. Penny, Walter Cocca, Marc W. Holderied, Lovasoa M.S. Rakotozafy, Franco Andreone

## References

[B1] AmphibiaWeb (2019) http://amphibiaweb.org/ [accessed on 15 October 2019]

[B2] Andreone F Rabibisoa N Randrianantoandro C Crottini A Edmonds D Kraus F Lewis JP Moore M Rabemananjara FCE Rabemanantoa JC Vences M (2014) Risk review is under way for invasive toad. Nature 512(7514): 253 10.1038/512253c 25143105

[B3] Andreone F Vences M Randrianirina JE (2001) Patterns of amphibian and reptile diversity at Berara Forest (Sahamalaza Peninsula), NW Madagascar. Italian Journal of Zoology 68: 235–241. 10.1080/11250000109356414

[B4] Avise JC Ball RM (1990) Principles of genealogical concordance in species concepts and biological taxonomy. In: Futuyma D Antonovics J (Eds) Surveys in Evolutionary Biology.Oxford University Press, New York, 45–67.

[B5] Birkinshaw CR (2004) Priority Areas for Plant Conservation. Ravintsara 2: 14–15.

[B6] Blommers-Schlösser RMA (1979) Biosystematics of the Malagasy frogs. I. Mantellinae (Ranidae). Beaufortia 29: 1–77. https://www.repository.naturalis.nl/document/548811

[B7] Blommers-Schlösser RMA Blanc CP (1991) Amphibiens (première partie). Faune de Madagascar 75(1): 1–384.

[B8] Bruford MW Hanotte O Brookfield JFY Burke T (1992) Single-locus and multilocus DNA fingerprinting. In: Hoelzel R (Ed.) Molecular genetic analysis of populations: a practical approach.IRL Press, Oxford, 225–269.

[B9] Castresana J (2000) Selection of conserved blocks from multiple alignments for their use in phylogenetic analysis. Molecular Biology and Evolution 17: 540–552. 10.1093/oxfordjournals.molbev.a026334 10742046

[B10] Crottini A Andreone F Edmonds D Hansen CM Lewis JP Rabemanantsoa JC Moore M Kraus F Vences M Rabemananjara F Randrianantoandro C (2014) A new challenge for amphibian conservation in Madagascar: the invasion of *Duttaphrynus melanostictus* in Toamasina province. FrogLog 111: 46–47.

[B11] Crottini A Glaw F Casiraghi M Jenkins RKB Mercurio V Randrianantoandro JC Randrianirina JE Andreone F (2011) A new Gephyromantis (Phylacomantis) frog species from the pinnacle karst of Bemaraha, western Madagascar. ZooKeys 81: 51–71. 10.3897/zookeys.81.1111 PMC3088064 21594161

[B12] Crottini A Madsen O Poux C Strauß A Vieites DR Vences M (2012) A vertebrate timetree elucidates the biogeographic pattern of a major biotic change around the K-T boundary in Madagascar. Proceedings of the National Academy of Sciences of the United States of America 109(14): 5358–5363. 10.1073/pnas.1112487109 22431616 PMC3325728

[B13] Darriba D Taboada GL Doallo R Posada D (2012) jModelTest 2: more models, new heuristics and parallel computing. Nature Methods 9(8): 772 10.1038/nmeth.2109 PMC4594756 22847109

[B14] De Sá RO Streicher JW Sekonyela R Forlani MC Loader SP Greenbaum E Richards S Haddad CFB (2012) Molecular phylogeny of microhylid frogs (Anura: Microhylidae) with emphasis on relationships among New World genera. BMC Evololutionary Biology 12: 241. 10.1186/1471-2148-12-241 PMC3561245 23228209

[B15] de Wit MJ (2003) Madagascar: heads it’s a continent, tails it’s an island. Annual Review of Earth and Planetary Sciences. 31: 213–248. 10.1146/annurev.earth.31.100901.141337

[B16] Dubos N Morel L Crottini A Freeman K Honoré J Lava H Nöel J Porton I Rendrirendry G Rosa GM Andreone F (2020) High interannual variability of a climate-driven amphibian community in a seasonal rainforest. Biodiversity and Conservation 29: 893–912. 10.1007/s10531-019-01916-3

[B17] Fouquet A Gilles A Vences M Marty C Blanc M Gemmell NJ (2007) Underestimation of species richness in Neotropical frogs revealed by mtDNA analyses. PLoS ONE 2: e1109. 10.1371/journal.pone.0001109 PMC2040503 17971872

[B18] Glaw F Köhler J Vences M (2012) A tiny new species of *Platypelis* from the Marojejy National Park in northeastern Madagascar (Amphibia: Microhylidae). European Journal of Taxonomy 9: 1–9. 10.5852/ejt.2012.9

[B19] Glaw F Vences M (2007) A field guide to the Amphibians and Reptiles of Madagascar. Third Edition. Vences & Glaw Verlags GbR, Köln, 496 pp.

[B20] Hall TA (1999) BioEdit: a user-friendly biological sequence alignment editor and analysis program for Windows 95/98/NT. Nucleic Acids Symposium Series 41: 95–98.

[B21] IUCN Standards and Petitions Committee (2019) Guidelines for using the IUCN Red List Categories and Criteria. Version 14. Prepared by the Standards and Petitions Committee. http://www.iucnredlist.org/documents/RedListGuidelines.pdf

[B22] IUCN (2019) The IUCN Red List of Threatened Species. Version 2019-2. http://www.iucnredlist.org [Downloaded on 18 October 2019]

[B23] Klages J Glaw F Köhler J Müller J Hipsley CA Vences M (2013) Molecular, morphological and osteological differentiation of a new species of microhylid frog of the genus *Stumpffia* from northwestern Madagascar. Zootaxa 3717: 280–300. 10.10.11646/zootaxa.3717.2.8 26176106

[B24] Köhler J Vences M D’Cruze N Glaw F (2010) Giant dwarfs: discovery of a radiation of large-bodied ‘stump-toed frogs’ from karstic cave environments of northern Madagascar. Journal of Zoology 282: 21–38. 10.10.1111/j.1469-7998.2010.00708.x

[B25] Kosuch J Vences M Dubois A Ohler A Böhme W (2001) Out of Asia: Mitochondrial DNA evidence for an oriental origin of tiger frogs, genus *Hoplobatrachus*. Molecular Phylogenetics and Evolution 21: 398–407. 10.1006/mpev.2001.1034 11741382

[B26] Kumar S Stecher G Tamura K (2016) MEGA7: Molecular Evolutionary Genetics Analysis, version 7.0 for bigger datasets. Molecular Biology and Evolution 33: 1870–1874. 10.1093/molbev/msw054 27004904 PMC8210823

[B27] Licata F Ficetola GF Freeman K Mahasoa RH Ravololonarivo V Fidy JFSN Koto-Jean AB Rasoanomenjanahary EN Andreone F Crottini A (2019) Abundance, distribution and spread of the invasive Asian toad *Duttaphrynus melanostictus* in eastern Madagascar. Biological Invasions 21(5): 1615–1626. https://link.springer.com/article/10.1007%2Fs10530-019-01920-2

[B28] Mittermeier RA Louis EE Richardson M Schwitzer C Langrand O Rylands AB Hawkins F Rajaobelina S Ratsimbazafy J Rasoloarison R Roos C Kappeler PM Mackinnon J (2010) Lemurs of Madagascar. 3^rd^ edition. Washington DC, Conservation International.

[B29] Padial JM Miralles A De la Riva I Vences M (2010) The integrative future of taxonomy. Frontiers in Zoology 7: 16. 10.1186/1742-9994-7-16 PMC2890416 20500846

[B30] Peloso PLV Frost DR Richards SJ Rodrigues MT Donnellan S Matsui M Raxworthy CJ Biju SD Lemmon EM Lemmon AR Wheeler WC (2016) The impact of anchored phylogenomics and taxon sampling on phylogenetic inference in narrow-mouthed frogs (Anura, Microhylidae). Cladistics 32: 113–140. 10.1111/cla.12118 34732021

[B31] Penny SG Andreone F Crottini A Holderied MW Rakotozafy LMS Schwitzer C Rosa GM (2014) A new species of the *Boophis rappiodes* group (Anura, Mantellidae) from the Sahamalaza Peninsula, Northwest Madagascar, with acoustic monitoring of its nocturnal calling activity. ZooKeys 435: 111–132. 10.3897/zookeys.435.7383 PMC4141189 25152689

[B32] Penny SG Andreone F Crottini A Holderied MW Rosa GM Schwitzer C (2016) The amphibians of the Sahamalaza Peninsula, northwest Madagascar – actions for their conservation. Bristol, United Kingdom, Bristol Zoological Society, 34 pp.

[B33] Penny SG Crottini A Andreone F Bellati A Rakotozafy LMS Holderied MW Schwitzer C Rosa GM (2017) Combining old and new evidence to increase the known biodiversity value of the Sahamalaza Peninsula, Northwest Madagascar. Contribution to Zoology 86(4): 273–296. 10.1163/18759866-08604002

[B34] Rakotoarison A Scherz MD Bletz MC Razafindraibe JH Glaw F Vences M (2019) Diversity, elevational variation, and phylogeographic origin of stump-toed frogs (Microhylidae: Cophylinae: *Stumpffia*) on the Marojejy Massif, northern Madagascar. Salamandra 55(2): 115–123.

[B35] Rakotoarison A Scherz MD Glaw F Köhler J Andreone F Franzen M Glos J Hawlitschek O Jono T Mori A Ndriantsoa SH Raminosoa N Riemann JC Rödel M-O Rosa GM Vieites DR Crottini A Vences M (2017) Describing the smaller majority: integrative taxonomy reveals twenty-six new species of tiny microhylid frogs (genus *Stumpffia*) from Madagascar. Vertebrate Zoology 67(3): 271–398.www.senckenberg.de/vertebrate-zoology [on 13.xi.2017]

[B36] Ralimanana H Ranaivojaona R (1999) Inventaire floristique et étude de la formation forestière dans la presqu’île Radama. Wildlife Conservation Society Madagascar, Unpublished report.

[B37] Rambaut A (2018) Tracer v1.7.1 https://github.com/beast-dev/tracer/releases/tag/v1.7.1 [accessed 15 September 2019]

[B38] Raselimanana AP (2008) Herpétofaune des forêts sèches malgaches. In: Goodman SM Wilmé L (Eds) Les forêts sèches de Madagascar.Malagasy Nature 1: 46–75.

[B39] Ronquist F Teslenko M van der Mark P Ayres DL Darling A Höhna S Larget B Liu L Suchard MA Huelsenbeck JP (2012) MrBayes 3.2: efficient Bayesian phylogenetic inference and model choice across a large model space. Systematic Biology 61(3): 539–542. 10.1093/sysbio/sys029 22357727 PMC3329765

[B40] Rozas J Ferrer-Mata A Sánchez-DelBarrio JC Guirao-Rico S Librado P Ramos-Onsins SE Sánchez-Gracia A (2017) DnaSP 6: DNA sequence polymorphism analysis of large datasets. Molecular Biology and Evolution 34: 3299–3302. 10.1093/molbev/msx248 29029172

[B41] Salzburger W Ewing GB Von Haeseler A (2011) The performance of phylogenetic algorithms in estimating haplotype genealogies with migration. Molecular Ecology 20: 1952–1963. 10.1111/j.1365-294X.2011.05066.x 21457168

[B42] Scherz MD Hutter CR Rakotoarison A Riemann JC Rödel M-O Ndriantsoa SH Glos J Hide Roberts S Crottini A Vences M Glaw F (2019) Morphological and ecological convergence at the lower size limit for vertebrates highlighted by five new miniaturised microhylid frog species from three different Madagascan genera. PLoS One 14: e0213314. 10.1371/journal.pone.0213314 PMC6436692 30917162

[B43] Scherz MD Vences M Rakotoarison A Andreone F Köhler J Glaw F Crottini A (2016) Reconciling molecular phylogeny, morphological divergence and classification of Madagascan narrow-mouthed frogs (Amphibia: Microhylidae). Molecular Phylogenetics and Evolution 100: 372–381. 10.1016/j.ympev.2016.04.019 27085671

[B44] Scherz MD Vences M Rakotoarison A Andreone F Köhler J Glaw F Crottini A (2017) Lumping or splitting in the Cophylinae (Anura: Microhylidae) and the need for a parsimony of taxonomic changes: a response to Peloso et al. (2017). Salamandra 53: 479–483.

[B45] Schwitzer N Randriatahina GH Kaumanns W Hoffmeister D Schwitzer C (2007) Habitat utilization of blue-eyed black lemurs, *Eulemur macaco flavifrons* (Gray, 1867), in primary and altered forest fragments. Primate Conservation 22: 79–87. 10.1896/052.022.0106

[B46] Schwitzer C Schwitzer N Randriatahina GH Rabarivola C Kaumanns W (2006) “Programme Sahamalaza”: New perspectives for the in situ and ex situ study and conservation of the blue-eyed black lemur (*Eulemur macaco flavifrons*) in a fragmented habitat. Proceedings of the German-Malagasy research cooperation in life and earth sciences 11: 135–149.

[B47] Stephens M Smith NJ Donnelly P (2001) A new statistical method for haplotype reconstruction from population data. American Journal of Human Genetics 68: 978–989. 10.1086/319501 11254454 PMC1275651

[B48] Van der Meijden A Vences M Hoegg S Boistel R Channing A Meyer A (2007) Nuclear gene phylogeny of narrow-mouthed toads (Family: Microhylidae) and a discussion of competing hypotheses concerning their biogeographical origins. Molecular Phylogenetics and Evolution 44: 1017–1030. 10.1016/j.ympev.2007.02.008 17369057

[B49] Vences M Glaw F Köhler J Wollenberg KC (2010) Molecular phylogeny, morphology and bioacoustics reveal five additional species of arboreal microhylids of the genus *Anodonthyla* from Madagascar. Contributions to Zoology 79: 1–32. 10.1163/18759866-07901001

[B50] Vences M Jovanovic J Glaw F (2008) Historical analysis of amphibian studies in Madagascar: an example for increasing research intensity and international collaboration. In: Andreone F (Ed.) A Conservation Strategy for the Amphibians of Madagascar.Monografie del Museo Regionale di Scienze Naturali di Torino 45: 47–58.

[B51] Vences M Raselimanana AP Glaw F (2003) Ranidae: *Hoplobatrachus*, Indian Tiger Frog. In: Goodman SM Benstead JP (Eds) The Natural History of Madagascar.The University of Chicago Press, Chicago and London, 926–927.

[B52] Vences M Vieites DR Glaw F Brinkmann H Kosuch J Veith M Meyer A (2003) Multiple overseas dispersal in amphibians. Proceedings of the Royal Society of London Series B Biological Sciences 270: 2435–2442. 10.1098/rspb.2003.2516 14667332 PMC1691525

[B53] Vieites DR Wollenberg KC Andreone F Köhler J Glaw F Vences M (2009) Vast underestimation of Madagascar’s biodiversity evidenced by an integrative amphibian inventory. Proceedings of the National Academy of Sciences 106: 8267–8272. 10.1073/pnas.0810821106 PMC2688882 19416818

[B54] Volampeno MSN (2009) Reproductive behaviour and habitat use in the blue-eyed black lemur (*Eulemur ﬂavifrons*, Gray, 1867) at the Sahamalaza Peninsula, National Park Madagascar. PhD thesis, Pietermaritzburg, South Africa: University of KwaZulu-Natal.

[B55] Volampeno MSN Masters JC Downs CT (2011) Life history traits, maternal behavior and infant development of blue-eyed black lemurs (*Eulemur flavifrons*). American Journal of Primatology 73: 474–484. 10.1002/ajp.20925 21254191

[B56] Wilmé L Goodman SM Ganzhorn JU (2006) Biogeographic evolution of Madagascar’s microendemic biota. Science 312(5776): 1063–1065. 10.1126/science.1122806 16709785

[B57] Wollenberg KC Vieites DR Van der Meijden A Glaw F Cannatella DC Vences M (2008) Patterns of endemism and species richness in Malagasy cophyline frogs support a key role of mountainous areas for speciation. Evolution 62(8): 1890–1907. 10.1111/j.1558-5646.2008.00420.x 18485110

[B58] Zimkus BM Lawson LP Barej MF Barratt CD Channing A Dash KM Dehling JM Preez LD Gehring P-S Greenbaum E Gvoždík V Harvey J Kielglast J Kusamba C Nagy ZT Pabijan M Penner J Rödel MO Vences M Lötters S (2017) Leapfrogging into new territory: how Mascarene ridged frogs diversified across Africa and Madagascar to maintain their ecological niche. Molecular Phylogenetics and Evolution 106: 254–269. https.//10.1016/j.ympev.2016.09.018 27664344

